# Testing the efficacy of web-based cognitive behavioural therapy for adult patients with chronic fatigue syndrome (CBIT): study protocol for a randomized controlled trial

**DOI:** 10.1186/s12883-015-0392-3

**Published:** 2015-08-12

**Authors:** Anthonie Janse, Margreet Worm-Smeitink, José Bussel-Lagarde, Gijs Bleijenberg, Stephanie Nikolaus, Hans Knoop

**Affiliations:** Expert Centre for Chronic Fatigue, Radboud university medical center, P.O. Box 9101, 6500 HB Nijmegen, The Netherlands

## Abstract

**Background:**

Cognitive behavioural therapy (CBT) is an effective treatment for fatigue and disabilities in patients with chronic fatigue syndrome (CFS). However, treatment capacity is limited. Providing web-based CBT and tailoring the amount of contact with the therapist to the individual needs of the patient may increase the efficiency of the intervention. Web-based CBT for adolescents with CFS has proven to be effective in reducing fatigue and increasing school attendance. In the proposed study the efficacy of a web-based CBT intervention for adult patients with CFS will be explored. Two different formats of web-based CBT will be tested. In the first format named *protocol driven feedback,* patients report on their progress and receive feedback from a therapist according to a preset schedule. In the second format named *support on demand*, feedback and support of the therapist is only given when patients ask for it. The primary objective of the study is to determine the efficacy of a web-based CBT intervention on fatigue severity.

**Method/Design:**

A randomized clinical trial will be conducted. Two-hundred-forty adults who have been diagnosed with CFS according to the US Centers for Disease Control and Prevention (CDC) consensus criteria will be recruited and randomized to one of three conditions: web-based CBT with *protocol driven feedback*, web-based CBT with *support on demand,* or wait list. Feedback will be delivered by therapists specialized in CBT for CFS. Each of the web-based CBT interventions will be compared to a wait list condition with respect to its effect on the primary outcome measure; fatigue severity. Secondary outcome measures are level of disability, physical functioning, psychological distress, and the proportion of patients with clinical significant improvement in fatigue severity. Outcomes will be assessed at baseline and six months post randomization. The web-based CBT formats will be compared with respect to the time therapists need to deliver the intervention.

**Discussion:**

As far as we know this is the first randomized controlled trial (RCT) that evaluates the efficacy of a web-based CBT intervention for adult patients with CFS.

**Trial registration:**

NTR4013

## Background

Chronic fatigue syndrome (CFS) i.e. often also named ME (myalgic encephalomyelitis/encephalopathy) by patient groups is characterised by medically unexplained, severe, and persisting fatigue that leads to substantial disability. Following the widely used US Centers for Disease Control and Prevention (CDC) consensus criteria for CFS, patients not only suffer from fatigue for six months or longer but also report four or more of eight additional symptoms: unrefreshing sleep, post-exertional malaise, headaches, muscle pain, sore throat, multi-joint pain, tender cervical or axillary lymph nodes, impaired short-term memory, and/or concentration problems [1, 2]. A recent meta-analysis comparing the prevalence rates of CFS from studies conducted in different countries found a mean prevalence rate of about 1 % [3]. Cognitive behavioural therapy (CBT) for CFS is an effective treatment [4], leading to a significant reduction of fatigue and disabilities. A subgroup of patients fully recovers following treatment [5, 6].

The cognitive behavioural model for CFS is based on the assumption that fatigue and disability are perpetuated by fatigue related beliefs and behaviours. CBT is aimed at changing these beliefs and behaviours. Individual CBT for CFS is an intensive treatment, requiring between 13 to 16 sessions over a period of six to eight months depending on the protocol used [7–10]. Treatment capacity and budgets for delivering treatment are limited. More efficient interventions are needed to both increase treatment capacity and reduce the costs of treatment. One way of achieving this is to develop a minimal intervention for the subgroup of CFS patients who do not need intensive CBT. Our research group developed one such a minimal intervention—guided self-instruction [11]. Guided self-instruction consists of a booklet with instructions combined with fortnightly email contact with a therapist. The information and instructions in the booklet are based on the protocol for individual CBT for CFS [8]. It has been repeatedly shown that guided self-instruction leads to a significant reduction of fatigue and disabilities [11–12]. For a subgroup of about 25 to 30 % of CFS patients the level of fatigue was within normal limits following the intervention. This is less than in face-to-face CBT. Guided self-instruction was also applied in a stepped care model for CFS. Stepped care started with guided self-instruction and, if insufficient, was followed by regular face-to-face CBT. Stepped care was as effective as face-to-face CBT but more time efficient as therapists had to invest less time per patient [13].

Although guided self-instruction is effective, fewer patients benefit from this intervention than from face-to-face therapy. Further development of guided self-instruction should be aimed at improving the efficacy of the intervention without substantially increasing the time therapists need to treat a patient. A possible way of achieving this is to design a web-based version of this intervention; CBIT (cognitive behavioural Internet therapy). The Internet has proved to be an effective medium for delivering CBT in a range of disorders [14]. Online therapy for CFS in adolescents—FITNET (Fatigue In Teenagers on the InterNET)—has proved to be as effective as face-to-face CBT in adolescent CFS patients [15].

Using online platforms for therapy offers new opportunities for interaction. Compared to the booklet used in guided self-instruction, online platforms provide more ways of communication, for example chat sessions or text-alerts via sms. Assignments can be interactive and patients can be given access to interview excerpts with patients that illustrate essential elements of the therapy. Therapists can get access to and offer feedback on the completed homework of patients so that they are able to follow the progress of the patient.

Although online CBT has shown to be effective in several disorders and also in adolescents with CFS, its efficacy in adult CFS patients has not yet been shown. We developed a web-based CBT for CFS based on the guided self-instruction and will test its efficacy in the proposed study. As little is known about the optimal form and amount of feedback delivered by the therapist [16], we developed two formats of the intervention.

In the first format, patients are asked by their therapist to report on their progress by email according to a schedule. In response, the therapist offers feedback. This format is named *protocol driven feedback*. Asking patients to email regularly and providing regular feedback according to a fixed schedule makes online CBT time consuming for therapists, possibly without increasing its efficacy. Patients may also report on their progress with respect to parts of the web-based CBT they do not need therapist input for. An alternative way of providing support is to tailor it to the needs of the patient and only give feedback when the patient asks for it. We assume that this will be more time efficient—i.e., requiring less therapist time to deliver, —than giving support according to the preset schedule. We named this second format of the web-based CBT *support on demand*. The efficacy of both web-based CBT interventions will be determined by comparing each web-based CBT format to a wait list control group with respect to its effect on the primary outcome measure: fatigue severity. Subsequently, we will determine if both forms of web-based therapy lead to a reduction of disability, improved physical functioning, less psychological distress and/or a higher proportion of patients with a clinically significant improvement in fatigue compared to a wait list condition. Furthermore, the effect on treatment outcome of how and when feedback is delivered to patients will be determined in an exploratory analysis by comparing the efficacy of the two web-based CBT formats with each other with respects to its effect on primary and secondary outcome measures. We will also compare therapists’ time needed to deliver the intervention. We hypothesize that:

1. Fatigue severity will be significantly lower at second assessment following web-based CBT compared to the wait list control group.
2. Patients who receive web-based CBT will report significantly less disabilities and psychological distress and significantly more often show clinical significant improvement in fatigue compared to the wait list control group.
3. Patients who received *protocol driven feedback* web-based CBT will report a significantly larger decrease in fatigue severity, level of disability, psychological distress and report more often a clinical significant improvement than patients who received web-based CBT with *support on demand*.

## Methods/Design

### Study design

**Fig. 1:**
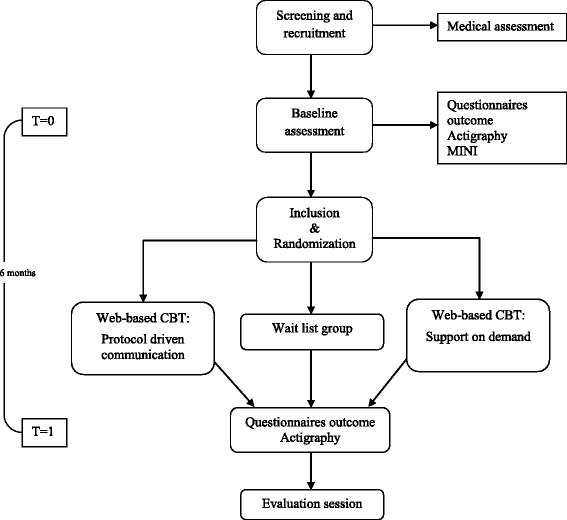
Flowchart of study

The efficacy of the web-based CBT will be determined in a randomized controlled trial (RCT) with a follow-up six months after randomization (T1). All patients will start with two intake sessions with a therapist and a baseline assessment (T0), which is part of clinical routine for CFS patients referred to our CFS treatment centre. At the second intake session, eligible patients will receive written information about the study from their therapist and will be asked to participate. Eligible patients who give written informed consent will be randomized into one of three conditions (see Fig. 1):

1. Web-based CBT with *protocol driven feedback*,
2. Web-based CBT with *support on demand* or
3. A wait list condition.

Patients in the wait list condition will wait for six months. After the evaluation session at T1, all patients from the wait list condition directly start with face-to-face CBT. They will not receive web-based CBT.

The primary endpoint of the study is fatigue severity at six months post-randomization (T1). Secondary outcome measures are level of disabilities, physical functioning, psychological distress, and proportion of patients with clinically significant improvement in fatigue. In the main analysis both conditions of the web-based CBT will be compared with a wait list condition with respect to the effect on fatigue severity. Subsequently, the effects of each of the treatment conditions on the secondary outcomes will be compared with the wait list control group.

#### Study population

In total 240 patients will participate in the study. All patients are referred to the Expert Centre for Chronic Fatigue (ECCF), a tertiary treatment centre for chronic fatigue of the Radboud university medical center in the Netherlands. All patients will meet CDC consensus criteria for CFS [1, 2]. They will all be severely fatigued with a score 35 or higher on the subscale fatigue of the Checklist Individual Strength (CIS). According to the consensus criteria the fatigue must be associated with significant disabilities. The latter is operationalized as scoring 700 or higher on the Sickness Impact Profile 8 (SIP8 total score). The other inclusion criteria are described in Table 1. Patients will be (temporarily) excluded when engaged in a legal procedure concerning disability-related financial benefits or when participating in other research [17]. We will assess the presence of psychiatric disorders using the MINI and clinical assessment and exclude patients who do not meet the inclusion criteria of the CDC with respect to current psychiatric disorders and/or having a medical history with psychiatric disorders.

**Table 1:** Inclusion and exclusion criteria

1) ≥ 18 years.	
2) Able to speak, read, and write Dutch.	
3) Able to use a computer and have access to Internet.	
4) CFS diagnosis according to the CDC consensus criteria:	[1, 2]
5) Severe fatigue is assessed with the subscale fatigue severity of the Checklist Individual Strength (CIS). Severe fatigue is operationalized as scoring ≥ 35.	[23]
6) Disabilities; Disability is assessed with the total score of the Sickness Impact Profile (SIP8). Severe disability is operationalized as a total score ≥ 700 on the SIP8.	[27, 41]
7) Given written informed consent.	
Exclusion criteria	
1) Engaged in a legal procedure concerning disability-related financial benefits.	
2) Participating in other CFS research.	

#### Ethical approval

This study will be carried out in accordance with The Code of Ethics of the World Medical Association (Declaration of Helsinki) for experiments that involve humans. This study has been reviewed and approved by the Medical Ethical Committee of the Radboud university medical center (reference NL42543.091.12) and has been registered in the Dutch Trial Register (trial number: NTR4013). Patients will receive verbal and written information about the study and will be asked to sign a written informed consent form before randomization and were requested not to follow other treatments for fatigue during participation in the study. Patients will receive written information about the study results when the paper reporting on the study has been published.

#### Randomization and blinding

Patients will be randomly allocated to one of the three conditions. This web-based randomization was computer-generated by creating blocks of 12 patients. Patients are contacted one week later to ask them if they want to participate in the study. An administrative assistant will call the patient and will perform randomization during this phone call. If patients do not need time to think about their decision and decide to participate, randomization will take place immediately at the second intake session. This will be performed by an administrative assistant in the presence of the therapist and patient. After randomization, therapists and patients will be able to read the randomization result on the screen i.e. ‘1) Internet therapy’ or ‘2) Internet therapy’ or ‘wait list’. Patients will not be informed that there are two web-based CBT treatment conditions. Knowledge of the two different treatment arms could affect the behaviour of the patient while following the web-based CBT. During the second intake session the therapist will explain that the therapist will be available to support them during the web-based CBT without specifying the frequency of support. In an instruction on the first page of the web-based application, only available after randomization, participants can read how to contact their therapist and when their therapist will contact them. The cognitive behavioural therapist who performs the intake will also deliver the web-based CBT. Test assistants will do all assessments. Therapists and test assistants are not blinded with respect to condition. This will most probably not introduce a bias as there is no contact between test assistants and patients following randomization. The test assistant sends a link via email to the patient, patients will fill in the questionnaires at home.

### Assessments

All patients have been medically examined prior to referral and somatic explanations for the fatigue will be ruled out either at the outpatient clinic of the department of internal medicine of the Radboud university medical center or by the referring general practitioner. The baseline assessment consists of two intake sessions and two test sessions at our treatment centre. Questionnaires are completed during both test sessions. Between test sessions, patients will wear an actometer and register their activities and symptoms in a diary. The actometer is a motion-sensing device worn at the ankle for 12 consecutive days and nights [18]. Trained cognitive behavioural therapists will perform the intake. They are all psychologists specialized in treating patients with CBT for CFS. During the first intake session with a therapist the Mini International Neuropsychiatric Interview (MINI) will be completed to assess the presence of psychiatric disorders [19]. At the half-year post randomization assessment, patients fill in the same questionnaires as at T0. Patients will also wear the actometer again and complete the diary. After randomization patients will receive a letter with the second assessment date and the face-to-face session with their therapist for evaluation of the web-based intervention or start of treatment for the wait list controls. Patients will receive reminders when questionnaires are not completed within one week. When patients do not want an evaluation session, the researcher will contact the patient with the request to complete the assessment as previously agreed. When a patient does not want to complete the full assessment, he or she will be requested to only fill in the questionnaire that assess the primary outcome measure.

We did not use a data monitoring committee for this study. All data will be monitored by a data-manager of the department who is not part of the research team. All participants have a study number in the data file. The file connecting study numbers with identifying personal data is separately stored and protected with a password that is only known to the principal investigator. All authors will have access to the final dataset.

### Intervention

Both web-based CBT interventions have a maximum duration of six months. All patients will receive a private username and password, ensuring private communication with their therapist. After patients accept the general terms of the web-based treatment, patients will get their unique username and password (by email) necessary for logging in on the portal.

The web-based CBT is based on the protocol for face-to-face CBT for CFS [8]. The protocol consists of several modules or subparts (see Table 2).

**Table 2:** Module titles and specific subparts of the web-based CBT for patients with CFS

Module title	Content of module
Getting started and goal setting	Psycho education about the cognitive-behavioural model of CFS and CBT, signing treatment contract and establish goals that if attained implies that a patient no longer has CFS.
Regulate sleep-wake cycle	Regulating sleep-wake cycle i.e., fixed bedtimes, no sleeping or lying down during the day
Helpful beliefs about fatigue	Formulate helpful beliefs
	Divert attention away from fatigue towards other activities and the environment
How to communicate with others about CFS	Change the communication about CFS with significant others
Gradually increasing my activities	Determine activity pattern
	Graded activity program for relative active patients (first spread activities evenly, followed by graded activity)
	Graded activity program for low active patient
	Pain: helpful beliefs for dealing with pain during graded activity
	Solve problems with the graded activity program
Reaching my goals step by step	Work resumption
	Increase mental activities
	Increase social activities
	Achieve my goals step by step
Evaluation and the future	Letting go of the rules of therapy (e.g., sleep in late, do a lot of activities from time to time)
	No longer being a CFS patient
	Having healthy levels of fatigue and learn how to stay healthy

After completion of the first *Getting started and goal setting*-module, the following modules—from ‘*Regulate sleep-wake cycle’* to ‘*Reaching my goals step by step’*—will be available to the patient. After completion of the sixth module, the last module ‘*Evaluation and the future’* will be available. Patients can send emails and have chat sessions with their therapists. Patients can also view interview excerpts with recovered CFS patients who share their experiences with web-based CBT. In these excerpts, patients explain the different elements of the treatment and talk about their experience with the intervention. Patients can print all texts and assignments. During development of the intervention, before the start of the study, seven patients were asked to evaluate the usability of the intervention. The think aloud method [20] was used in this pilot and improvements have been made to the Internet program. Three out of these seven patients were interviewed. Excerpts of these interviews were reported in this trial paper. After the pilot study, the intervention will only be available to patients participating in the study and allocated to one of the treatment arms. Therapists have access to the assignments and registration forms that are filled in by patients. In a box at the bottom of each completed assignment therapists can give feedback. In both conditions the therapist will respond within five working days after the patient sent an email. When practical problems with the program occur or when communication between therapist and patient is not sufficient to resolve a specific problem via email or by chatting, telephone contact will be offered to the patient. All data of patients—i.e., assignment, forms, and communications with the therapist—will be encrypted and securely stored on the mainframe of the Internet portal supplier.

#### Low active versus relative active

With data from the actometer, two activity patterns can be discerned: a low activity pattern and a relatively active pattern. The individuals' activity patterns will be based on the 12 daily physical activity scores (van der Werf, Prins, Vercoulen, van der Meer, & Bleijenberg, 2000). Two physical activity patterns can be discerned. The average daily physical activity scores of low active patients stay below the general mean physical activity of CFS patients in at least 11 of 12 days. Relative active patients score at least 2 of 12 days above the mean physical activity score of CFS patients. Relative active patients have fluctuating activity levels with bursts of activity followed by (prolonged) periods of rest [18]. In case of missing actometer data at baseline, therapists will determine the activity pattern by using a structured interview [21].

In accordance with the CBT protocol for face-to-face therapy, we developed a low active and a relatively active variant of the two web-based CBT formats. Low active patients will start with a graded activity program early in therapy. Relatively active patients will first learn to spread their activities more evenly before starting the graded activity program. Following Stulemeijer [22] we do not expect different treatment outcomes for low or relatively active patients.

#### Web-based CBT—protocol driven feedback

Patients following web-based CBT with *protocol driven feedback* will be asked by the therapist to report on their progress according to a schedule set by the therapist. The therapist asks the patient to email at least weekly in the first four weeks and once every two weeks in the next eight weeks. After this period the therapist will propose a schedule dependent on the progress being made. We expect this will likely be once every two or three weeks until the end of the program. Therapists will send reminders if patients do not follow the schedule. Patients can book chat sessions with their therapists. Phone support will only be provided when needed urgently.

#### Web-based CBT—support on demand

Patients who will follow the web-based CBT with *support on demand* will only receive feedback if they ask for it. Patients will not receive any reminders from the therapist if they do not report on their progress via email.

#### Training and supervision of therapists

The therapists delivering the web-based CBT are psychologists and cognitive behavioural therapists who are trained and experienced in delivering CBT for CFS. They will receive weekly group supervision regarding the web-based CBT. All therapists received training to improve their online communication skills with patients. The therapist will aim to write the emails in such a way that it motivates patients to follow the instructions of the intervention.

### Adherence, drop-out, and treatment integrity

Patients are assumed to have started with the treatment after they logged in on the Internet portal three times or more and have established goals for therapy on the goal sheet of the first module. We will assess how patients have used the web-based CBT by registration of 1) the number of times logged on; 2) the total duration of all sessions in minutes; 3) the number of opened treatment modules 4) and the number of sent emails and chat sessions. These variables will be reported.

We assume that a patient adheres to the web-based CBT with *protocol driven feedback* if: 

- He or she at least fortnightly emailed to the therapist, and
- has opened at least each module of the web-based intervention once.

We assume that a patient adheres to the web-based CBT with *support on demand* if:

- They have opened at least each module of the web-based intervention once.

Treatment integrity will be determined by evaluating a random selection of five percent of the emails send by the therapist. Two experienced therapists (HK; AJ) will independently score to what extent the feedback given to the patients is according to the protocol for CBT for CFS (Knoop & Bleijenberg, 2010). We will register to what extent therapists have followed the preset schedule in the ‘*protocol driven feedback’* condition.

#### Outcomes

**Fig. 2:**
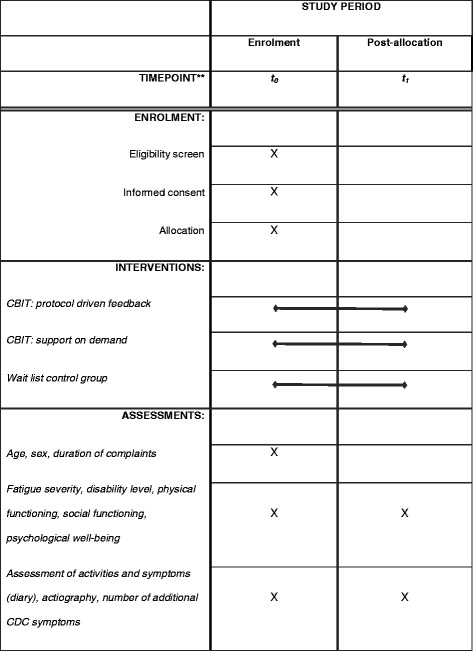
Schedule of enrolment, interventions, and assessments

All outcome measures are listed in Table 3. See Fig. 2 for a schedule of enrolment, interventions and assessments.

**Table 3:** Outcome measures

	*Instruments*
Primary outcome measure	
Fatigue severity	Checklist Individual Strength, (CIS) subscale fatigue severity
Secondary outcome measures	
Level of disabilities	Sickness Impact Profile-8, (SIP8) total score
Physical functioning	Medical Outcomes Survey Short Form-36, (SF-36) subscale physical functioning
Psychological distress	Symptom Checklist 90items, (SCL-90) total score
Clinical significant improvement in fatigue	Checklist Individual Strength, (CIS) subscale fatigue severity < 35 and a reliable change index > 1.96

#### Primary outcome measure

Fatigue severity will be assessed with the subscale fatigue severity of Checklist Individual Strength [23]. This subscale consists of eight items assessing fatigue severity over the past two weeks. Scores range between eight (*no fatigue*) and 56 (*severe fatigue*). The cut-off score for severe fatigue is 35. This is two standard deviations above the mean of healthy controls [24]. The CIS is a reliable and valid instrument for the assessment of fatigue in CFS patients [23–25]. The Cronbach's alpha reliability coefficient for the subscale fatigue severity is .88 [23]. This outcome measure and cut-off point was also used in previous trials assessing the efficacy of a minimal intervention for CFS [26].

#### Secondary outcome measures

Level of disability will be measured with the total score of the Sickness Impact Profile (SIP8) [25]. The SIP8 assesses functional disability on the following eight domains: ambulation, home management, mobility, alertness behaviour, sleep and rest, work, social interactions, and recreation and pastimes. A weighted total score is computed from the scores on the eight subscales [12, 27]. This widely used measure has good reliability [27] and validity [5, 11].

Physical functioning will be assessed with the Medical Outcomes Survey Short Form-36 (SF-36) [28]. The subscale ‘physical functioning’ will be used. Scores on both scales range from 0 (maximum limitations) to 100 (no limitations). The SF-36 is a reliable and valid instrument to assess self-reported health status in CFS patients [28, 29].

Psychological distress will be assessed with the total score of the Symptom Checklist 90 (SCL-90) [30]. In total 90 items are answered on a five-point Likert scale. Total scores range from 90 to 450, with higher scores being indicative of more psychological distress. This widely used measure has good reliability and discriminating validity [31].

Clinical significant improvement in fatigue is defined as a reliable change index > 1.96 [32] and a score of < 35 on the CIS ‘fatigue severity’ subscale at second assessment.

#### Other study parameters

Based on the CBT for CFS model several fatigue related behaviours and cognitions will be assessed [33, 34]. The therapist will register invested therapist time for each patient. Actigraphy will be used to assess the level of physical activity. The actometer has been shown to be a reliable and valid instrument for the assessment of physical activity in CFS [18].

The presence of psychiatric disorders will be assessed using a structured diagnostic interview, the Mini International Neuropsychiatric Interview (MINI) screen test [19].

#### Adverse events

Adverse events will be assessed six months post randomization. Patients will be asked if they experienced new symptoms or an increase of existing symptoms during therapy or wait period. Patients who have received treatment will be asked if therapy in their opinion had negative side effects. Previous treatment studies have shown that regular face-to-face CBT and guided self-instructions for CFS are safe [35, 36].

### Statistical analysis

To determine whether web-based CBT leads to a reduction of fatigue severity compared to a wait list condition ANCOVA will be used. The second assessment of the primary outcome measure fatigue severity is the dependent variable in this analysis, with baseline CIS score as covariate, and condition as fixed factor. In RCTs ANCOVA yields greater power than other statistical methods [37]. Analyses will be based on intention to treat. Multiple imputation using fully conditional specification will be used to handle missing observations. The number of imputations will be at least equal to the percentage of missing data of the outcome measure. The assumption is made that data are missing at random [38]. A priori contrasts will be defined for the factor condition comparing web-based CBT with *protocol driven feedback* versus wait list and *support on demand* versus waiting list. When statistical significant differences are found, a sensitivity analysis will be performed on the basis of different assumptions about the values of missing data.

For the secondary outcome measures disabilities, physical functioning, psychological distress and the proportion of patients with clinical significant improvement in fatigue severity, the same analysis will be repeated, but with the secondary outcome measures at the second assessment as dependent variable, and the scores of these measurements at baseline as covariate. Statistical significance will be assumed at p < 0.025 (.05 corrected for two comparisons: web-based CBT with protocol driven feedback versus waiting list and web-based CBT with support on demand versus wait list) for the analysis of the results with respect to the primary outcome measure fatigue and p < 0.05 for secondary outcome measures. We will use a chi-square test to determine if the proportion of patients with a clinical improvement outcome significantly differ between treatment arm and wait list condition. We will also use ANCOVA to explore possible differences between the two web-based CBT formats with fatigue severity as dependent variable, baseline score as covariate and condition as fixed factor. Mean therapist time needed per patient in each web-based CBT format will be compared with an independent samples *t*-test and using bootstrapping when the therapist times are too skewed.

### Sample size calculation

Based on a previous study with guided self-instructions [11] we expect a mean difference on the CIS fatigue severity score between each web-based CBT format and the wait list condition of 6.7. The standard deviation in this study at second assessment was 12.1 in the treatment condition and 8.7 in the wait list condition. In order to answer the primary objective of this study to detect a difference of 6.7 points on the CIS fatigue between each of the web-based CBT formats and the wait list condition, assuming the same standard deviations as in the aforementioned study, with a two sided alpha of .025 and a, power of .95, 76 patients are needed in each of the three conditions at T1. This number is based on a *t*-test. Because an ANCOVA with baseline assessment as covariate will be used (which increases statistical power) this sample size has been multiplied with (1–0.342^2^) [39]. The correlation between the baseline CIS-fatigue score and the score at second assessment was 0.342 in the aforementioned study. After correction 68 patients with complete data per condition are needed. Assuming a drop-out rate of 15%, based on previous trials testing the guided self-instruction [11, 12] we will have to include 80 patients in each group to have 95% power to detect the expected difference of 6.7 points on the CIS fatigue between each of the CBT formats and the wait list condition.

In an exploratory analysis we will determine whether there is a difference in efficacy between web-based CBT with *protocol driven feedback* and web-based CBT with *support on demand*. On the basis of the calculated sample size of 68 patients with complete data per treatment arm, a power of .80 and a two sided significance level of 0.05 we will be able to detect a difference between both conditions that corresponds with an effect size (Cohen’s d) of 0.48 (moderate size effect size).

## Discussion

The CBIT trial outlined in this article will be the first randomized clinical trial testing the efficacy of a web-based CBT for adults with CFS. It will determine if web-based CBT leads to a significant reduction of fatigue. Secondary outcome measures are disabilities, physical functioning, psychological distress, and clinically significant improvement in fatigue severity. If this CBIT study shows that online CBT is an effective treatment for severe fatigue and disability, it can be a first choice treatment for patients with CFS as web-based interventions can reach also those patients who are unable to visit our treatment centre due to geographical location or other circumstances that complicate face-to-face interventions in an outpatient clinic. The web-based CBT will be time efficient for patients. Therapists can be more flexible in their daily scheduling as there are no face-to-face sessions with a fixed timeslot.

In an exploratory analysis we will determine whether there is a difference in efficacy when patients receive *support on demand* or when they interact with the therapist according to a schedule. However, given the relatively small sample size a considerable difference between both conditions has to be present in order to detect it. Information about the amount of time of the therapist needed to deliver the two formats of web-based CBT could have implications for the decision which of the web-based interventions could best be implemented in clinical practice.

This study has some potential limitations. We have no controlled follow-up assessment in our study. We will not be able to determine if the expected positive effects of the web-based intervention are sustained over a longer period. A longer follow-up period is not possible as our study will be continued as a randomized noninferiority trial comparing the two forms of web-based CBT followed by face to face CBT if patients have not profited from the internet intervention (stepped care) with care as usual, i.e. face to face CBT following the waiting list. This randomized noninferiority trial is registered in the Netherlands trial register (NTR4809). Second potential limitation is that the treatment effects cannot be controlled for non-specific factors of the interventions. As this study will be continued as a randomized controlled noninferiority trial an active control was not possible. Previous research has shown that CBT is significantly more effective than other active interventions, like guided support groups and specialist medical care (Prins et al., 2001 and White et al., 2011).

Although we expect that the web-based CBT will be more effective than guided self-instruction, it is possible that some patients do not profit from web-based CBT and they could benefit from additional face-to-face CBT. In a follow-up study we will test whether stepped care, consisting of a form of web-based CBT followed by individual CBT if a patient has not benefited from the web-based CBT, is as effective but more efficient than face-to-face CBT. The results of this follow-up study could help to further broaden treatment possibilities for adults with CFS.

## Abbreviations

ANCOVA: Analysis of covariance
CBIT: Cognitive behavioural internet therapy
CBT: Cognitive behavioural therapy
CFS: Chronic fatigue syndrome
ME: Myalgic encephalomyelitis/encephalopathy
CDC: U.S. Centers for disease control and prevention
